# Higher Life’s Crucial 9 protects against infertility among U.S. women aged 18–45 years

**DOI:** 10.3389/fendo.2025.1465022

**Published:** 2025-01-30

**Authors:** Shaoqun Huang, Shuqin Duan, Seok Choi, Hongyang Gong

**Affiliations:** ^1^ Department of Oncology Surgery, Fuzhou Hospital of Traditional Chinese Medicine Affiliated to Fujian University of Traditional Chinese Medicine, Fuzhou, Fujian, China; ^2^ Department of Obstetrics and Gynaecology, The Second Hospital of Jilin University, Changchun, Jilin, China; ^3^ Department of Physiology, College of Medicine, Chosun University, Gwangju, Republic of Korea

**Keywords:** infertility, Life’s Crucial 9, association, National Health and Nutrition Examination Survey, cardiovascular health

## Abstract

**Objective:**

Infertility is not only a reproductive issue but is also closely linked to cardiovascular health and other factors. Life’s Crucial 9 (LC9) is a set of lifestyle guidelines aimed at improving cardiovascular health, yet its potential association with infertility remains underexplored. This study aims to investigate the relationship between LC9 and infertility, providing new insights and strategies for the prevention and management of infertility.

**Methods:**

This study utilized data from the National Health and Nutrition Examination Survey (NHANES) from 2013 to 2018. Multivariate logistic regression and weighted quantile sum (WQS) regressions were employed to investigate the association between LC9 and infertility. Restricted cubic spline (RCS) analysis explored the linear or non-linear relationship between LC9 and infertility. Interaction analyses were conducted on subgroups to validate the findings.

**Results:**

There was a significant negative association between LC9 and infertility. After adjusting for covariates, for every 10-point increase in LC9, there was a 35% decrease in the prevalence of infertility (*P* < 0.001). This negative correlation persisted when LC9 was divided into quartiles. Moreover, as LC9 increased, there was a trend towards lower infertility prevalence (*P for trend* < 0.001). WQS analyses showed consistent associations (OR=0.27, 95%CI: 0.14, 0.53), with sleep health score, psychological health score, and Body mass index score as significant factors. The dose-response curve indicated a linear association between LC9 and infertility, with higher LC9 associated with lower infertility risk.

**Conclusion:**

The results of this study show a strong negative correlation between LC9 and the prevalence of infertility. Clinically, these findings offer hope for infertility patients, suggesting that adherence to a higher LC9 score significantly reduces the risk of infertility. This will provide a new avenue for infertility prevention and management, offering hope and potential relief to infertile patients.

## 1 Introduction

Infertility is defined as the inability to conceive after at least 12 months of unprotected intercourse. Approximately 8%-12% of couples of reproductive age worldwide suffer from infertility (1). Although male infertility accounts for more than half of these cases, the burden of infertility is still predominantly borne by women (2). Infertility is a condition characterized by reproductive dysfunction resulting from various etiologies and is a significant reproductive health issue for couples of childbearing age. The causes of infertility can be attributed to organic factors (such as pelvic diseases) or iatrogenic factors (such as postoperative adhesions), but lifestyle and psychological stress may also play a role (3). The risk factors and mechanisms of infertility remain subjects of ongoing research. Several studies have indicated that infertility is associated with sleep (3), diet (4), appropriate exercise (5), diabetes (6), obesity (7), dyslipidemia (8), cardiovascular diseases (9), and smoking (10).

In 2010, the American Heart Association (AHA) defined cardiovascular health (CVH) and quantified it using seven metrics known as Life’s Simple 7, which includes diet, physical activity, smoking status, BMI, total cholesterol, blood pressure, and blood glucose. In 2022, the AHA added sleep as a new component of CVH, introducing Life’s Essential 8 (LE8) to further encourage personal and community health and to provide a more comprehensive reflection of cardiovascular health (11). On February 27, 2024, an article published in Circulation highlighted that individual mental health is a crucial component of enhancing cardiovascular health (12). Therefore, it is proposed to expand Life’s Essential 8 (LE8) by adding a mental health metric, creating what can be termed Life’s Crucial 9 (LC9). LC9 encompasses four health behaviors (diet, physical activity, smoking cessation, sleep) and five health factors (healthy weight, normal cholesterol levels, blood glucose, blood pressure, and mental health). Compared to LE8, LC9 redefines cardiovascular health by incorporating the new mental health metric, making it more aligned with clinical practice. Consequently, LC9 can better represent an individual’s cardiovascular health status and more accurately reflect the underlying pathophysiological changes within the body.

To date, numerous studies have explored the impact of the four health above behaviors and five health factors of CVH on infertility. However, the relationship between LC9 (the composite metric) and infertility has not yet been investigated. Previous research has indicated that infertility is associated with lower CVH scores in middle-aged women, focusing solely on diet, sleep, physical activity, smoking, BMI, total cholesterol, blood pressure, and blood glucose (13), without considering the impact of mental health on infertility. Nevertheless, mental health is very prevalent in the occurrence and development of infertility (14–19), and various psychological interventions are currently employed to address infertility (20).

With the ongoing changes in global lifestyles and environmental factors, the incidence of infertility has shown a marked upward trend. Given its complex etiology and uncertain treatment options, identifying modifiable factors associated with infertility has become a critical focus of current research, aiming to intervene and prevent its occurrence. Therefore, this study utilized data from the 2013-2018 cycles of the National Health and Nutrition Examination Survey (NHANES) in the United States. This database, with its comprehensive individual health indicators and lifestyle data, provides a broad population sample and a solid foundation for investigating infertility-related factors and their impact on cardiovascular health. For the first time, this study systematically analyzed the relationship between various independent LC9 factors and infertility, evaluating their potential roles in preventing infertility. This research not only aims to uncover further details about the complex causes of infertility but also provides important evidence-based support for clinical practice, advancing the development and implementation of public health strategies.

## 2 Methods

### 2.1 Study population

**Figure 1:**
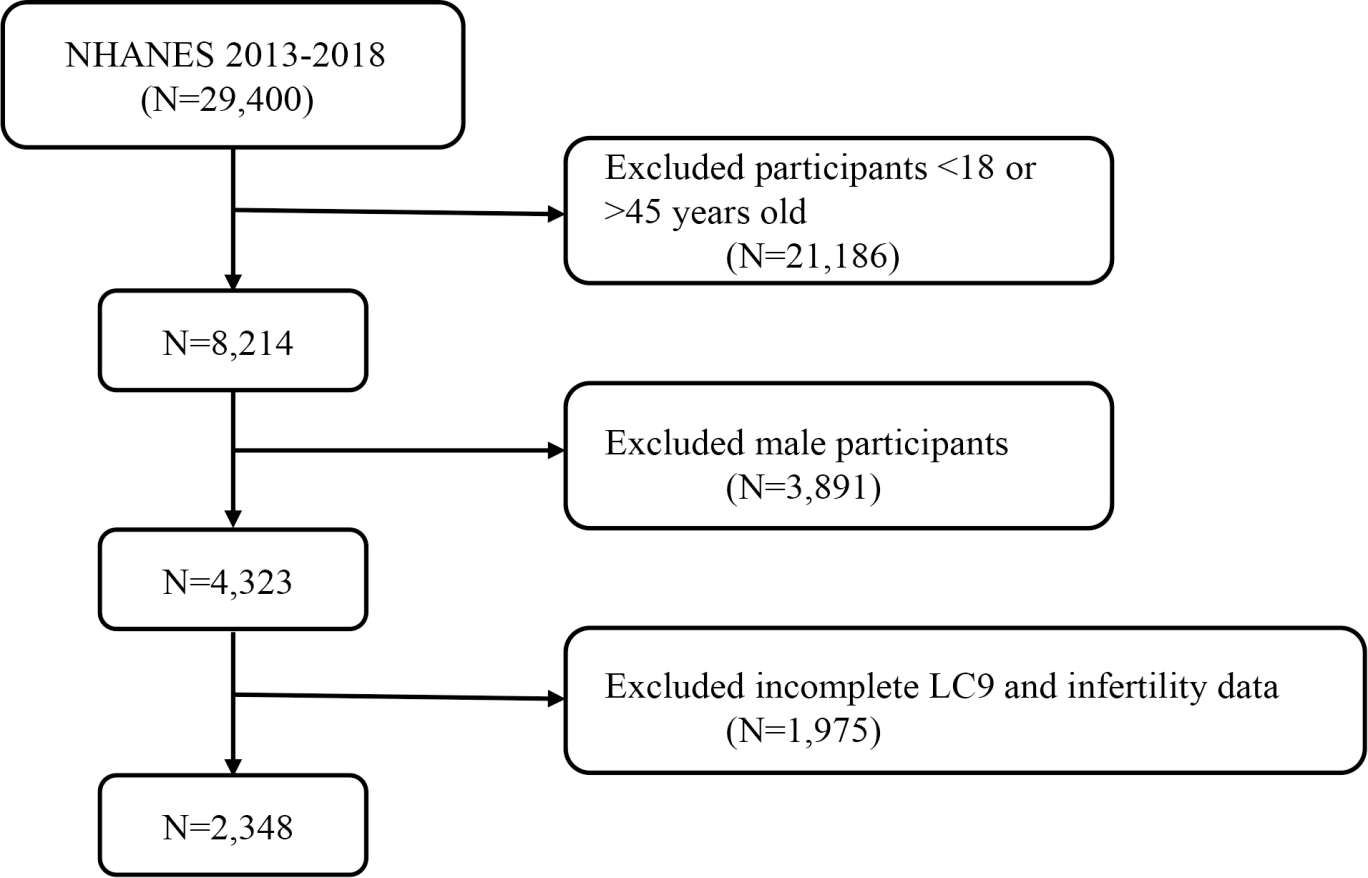
A flow diagram of eligible participant selection in the National Health and Nutrition Examination Survey.

The National Health and Nutrition Examination Survey (NHANES) is a nationally representative cross-sectional survey conducted through home interviews and mobile examination centers, aimed at assessing the health and nutritional status of the U.S. population (21). This survey utilized data from 29,400 participants over three cycles of NHANES, spanning from 2013 to 2018. After excluding individuals <18 or >45 years old (n = 21,186), males (n = 3,891), and participants with missing or incomplete LC9 and infertility data (n = 1,975), a total of 2,348 participants were included in the final analysis. **Figure 1** displays a flowchart of the entire selection process. NHANES is approved by the Research Ethics Review Board of the National Center for Health Statistics, and all participants provided informed consent (22). The data used in this study are de-identified and publicly available (https://www.cdc.gov/nchs/nhanes/index.htm).

### 2.2 Measurement

#### 2.2.1 Assessment of Life’s Crucial 9

LC9 includes nine metrics: four health behaviors (healthy diet, physical activity, smoking cessation, and healthy sleep) and five health factors (weight management, cholesterol control, blood glucose management, blood pressure management, and mental health). Detailed instructions on calculating each participant’s LC9 score using the NHANES database can be found in **Supplementary Table S1**. In summary, each of the nine LC9 metrics has a score ranging from 0 to 100. The overall LC9 score is determined by averaging the scores of the nine individual metrics. A healthy diet is assessed using the Healthy Eating Index 2015 (HEI-2015) (23, 24). The components and scoring criteria of HEI-2015 are detailed in **Supplementary Table S2**. Sleep health, smoking status, physical activity, and mental health are derived from standardized questionnaires, while BMI, blood pressure, blood glucose, and cholesterol are obtained by trained professionals from the NHANES database (https://www.cdc.gov/nchs/nhanes/index.htm).

#### 2.2.2 Diagnosis of infertility

As in previous studies (25, 26), infertility is defined as a decrease in the ability of an individual or their partner to conceive, evidenced by the inability to achieve pregnancy after one year or more of regular, unprotected intercourse. In this study, the assessment of infertility was derived from the NHANES Reproductive Health Questionnaire (RHQ074). Specifically, participants were asked the following question: “Have you ever tried to become pregnant for a year or longer without becoming pregnant?” Those who answered “yes” were classified as infertile (https://wwwn.cdc.gov/Nchs/Nhanes/2013-2014/RHQ_H.htm#RHQ074).

### 2.3 Covariables

According to previous studies (25, 26), the covariates in this research include age, race, marital status, education level, family poverty-to-income ratio (PIR), smoking, alcohol consumption, Physical activity, hypertension, diabetes, and hypercholesterolemia. For detailed information on these covariates, please refer to **Supplementary Table S3**.

### 2.4 Statistical analyses

In this study, all data were statistically analyzed using R (version 4.3.1). The data were weighted, with continuous variables presented as mean ± standard deviation, and p-values calculated using weighted linear regression models. Percentages for categorical variables (weighted N, %) and their p-values were calculated using weighted chi-square tests. The association between LC9 and infertility was analyzed using multivariable logistic regression models, where LC9 was categorized into quartiles. Trend tests and p-values for linear trends were calculated to determine the consistency of the relationship. Three models were constructed in this study: (1) an unadjusted crude model; (2) a model adjusted for age, race, education level, marital status, and family poverty-to-income ratio (PIR); and (3) a model further adjusted for smoking, alcohol consumption, Physical activity, hypertension, diabetes, and hypercholesterolemia. A smooth curve fitting was applied to further explore the potential linear relationship between LC9 and infertility. Additionally, odds ratios (ORs) were calculated for every 10-point increase in LC9, with subgroup analyses conducted based on age, race, marital status, education level, PIR, smoking, physical activity, alcohol consumption, hypertension, diabetes, and hypercholesterolemia.

In addition, we applied the WQS to explore the overall effect of each LC9 metric on infertility. LC9 metric with a WQS weighting (ranging from 0 to 1 and summing to 1) higher than 0.111 (the mean of the 9 LC9 metric) were identified as major contributors. The significance was determined by p-values below 0.05.

## 3 Results

### 3.1 Characteristics of the participants

This study included 2,348 women aged 18 to 45, representing approximately 36.26 million reproductive-aged women in the United States. The prevalence of infertility was 13% (equivalent to 4.66 million), with an average (SD) CVH score of 70.74 (14.23). **Table 1** (weighted) indicates that compared to women without infertility, those with infertility had lower CVH scores [no infertility: 76.62 (13.29); infertility: 70.74 (14.23)]. Statistically significant differences (all p<0.05) were observed between infertility and non-infertility groups in terms of age, cohabitation status, smoking, alcohol consumption, hypertension, diabetes, and hyperlipidemia. Compared to women without infertility, those with infertility tended to be older (35-45 years), married, Caucasian, and heavy drinkers. The unweighted baseline characteristics are detailed in **Supplementary Table S4**.

**Table 1:** Baseline characteristics of all participants were stratified by infertility, weighted.

Characteristic	Overall, N = 36,266,192 (100%)	Non-infertility, N = 31,597,899 (87%)	Infertility, N = 4,668,293 (13%)	P Value
**No. of participants in the sample**	2,348	2,065	283	**-**
**Age (%)**				**<0.001**
* 18-25*	8,475,204 (23%)	7,935,345 (25%)	539,859 (12%)	
* 26-34*	11,943,370 (33%)	10,579,120 (33%)	1,364,250 (29%)	
* 35-45*	15,847,619 (44%)	13,083,435 (41%)	2,764,184 (59%)	
**Race (%)**				0.153
* Non-Hispanic White*	20,817,235 (57%)	17,847,849 (56%)	2,969,386 (64%)	
* Other*	6,503,832 (18%)	5,865,240 (19%)	638,592 (14%)	
* Non-Hispanic Black*	4,655,868 (13%)	4,104,606 (13%)	551,263 (12%)	
* Mexican American*	4,289,256 (12%)	3,780,205 (12%)	509,052 (11%)	
**Married/live with partner (%)**				**<0.001**
* No*	14,416,365 (40%)	13,408,319 (42%)	1,008,046 (22%)	
* Yes*	21,849,827 (60%)	18,189,580 (58%)	3,660,247 (78%)	
**Education level (%)**				0.971
* Below high school*	3,785,024 (10%)	3,300,393 (10%)	484,631 (10%)	
* High School or above*	32,481,168 (90%)	28,297,507 (90%)	4,183,662 (90%)	
**PIR (%)**				0.260
* Not Poor*	24,693,013 (72%)	21,283,046 (72%)	3,409,967 (75%)	
* poor*	9,400,423 (28%)	8,281,507 (28%)	1,118,915 (25%)	
**Smoking (%)**				**0.048**
* Never*	24,972,694 (69%)	22,049,273 (70%)	2,923,421 (63%)	
* Former*	4,638,919 (13%)	3,997,170 (13%)	641,749 (14%)	
* Current*	6,654,580 (18%)	5,551,457 (18%)	1,103,123 (24%)	
**Physical activity (%)**				0.470
* Inactive*	4,284,392 (15%)	3,706,519 (14%)	577,874 (16%)	
* Active*	25,233,812 (85%)	22,243,259 (86%)	2,990,553 (84%)	
**Drinking (%)**				**0.047**
* former*	1,741,402 (5.0%)	1,350,142 (4.4%)	391,260 (8.7%)	
* heavy*	9,055,486 (26%)	7,731,973 (25%)	1,323,513 (29%)	
* mild*	9,565,997 (27%)	8,453,627 (28%)	1,112,371 (25%)	
* moderate*	10,241,369 (29%)	9,041,706 (30%)	1,199,663 (27%)	
* never*	4,539,156 (13%)	4,069,652 (13%)	469,504 (10%)	
**Hypertension (%)**				**0.001**
* No*	30,640,616 (84%)	27,036,788 (86%)	3,603,829 (77%)	
* Yes*	5,625,576 (16%)	4,561,112 (14%)	1,064,465 (23%)	
**Diabetes (%)**				**0.007**
* No*	34,396,392 (95%)	30,134,993 (95%)	4,261,399 (91%)	
* Yes*	1,869,800 (5.2%)	1,462,907 (4.6%)	406,894 (8.7%)	
**High cholesterol (%)**				**0.007**
* No*	31,460,705 (87%)	27,690,895 (88%)	3,769,810 (81%)	
* Yes*	4,805,488 (13%)	3,907,004 (12%)	898,484 (19%)	
**Mean LC9 score (mean (SD))**	75.86 (13.56)	76.62 (13.29)	70.74 (14.23)	**<0.001**
**Mean HEI-2015 diet score (mean (SD))**	37.78 (31.99)	38.44 (32.15)	33.35 (30.56)	**0.048**
**Mean physical activity score (mean (SD))**	76.88 (39.10)	77.54 (38.64)	72.42 (41.93)	0.148
**Mean tobacco exposure score (mean (SD))**	75.86 (38.95)	76.62 (38.43)	70.68 (42.01)	**0.039**
**Mean sleep health score (mean (SD))**	84.63 (23.22)	85.05 (22.95)	81.76 (24.84)	0.088
**Mean psychological health score (mean (SD))**	86.84 (25.63)	87.50 (24.84)	82.35 (30.11)	**0.037**
**Mean body mass index score (mean (SD))**	59.59 (37.11)	61.17 (36.58)	48.84 (38.96)	**0.002**
**Mean blood lipid score (mean (SD))**	80.25 (26.29)	80.90 (25.88)	75.87 (28.59)	**0.027**
**Mean blood glucose score (mean (SD))**	93.80 (16.76)	94.46 (15.93)	89.30 (21.03)	**<0.001**
**Mean blood pressure score (mean (SD))**	87.14 (22.34)	87.89 (21.90)	82.04 (24.57)	**0.002**

Mean (SD) for continuous variables: the P value was calculated by the weighted linear regression model.

Percentages (weighted N, %) for categorical variables: the P value was calculated by the weighted chi-square test.

LC9, Life’s Crucial 9; CVH, cardiovascular health; PIR, Ratio of family income to poverty; ORs, odds ratios; CI, confidence interval.

The bold values are less than 0.05.

### 3.2 Association between LC9 and infertility

**Figure 2:**
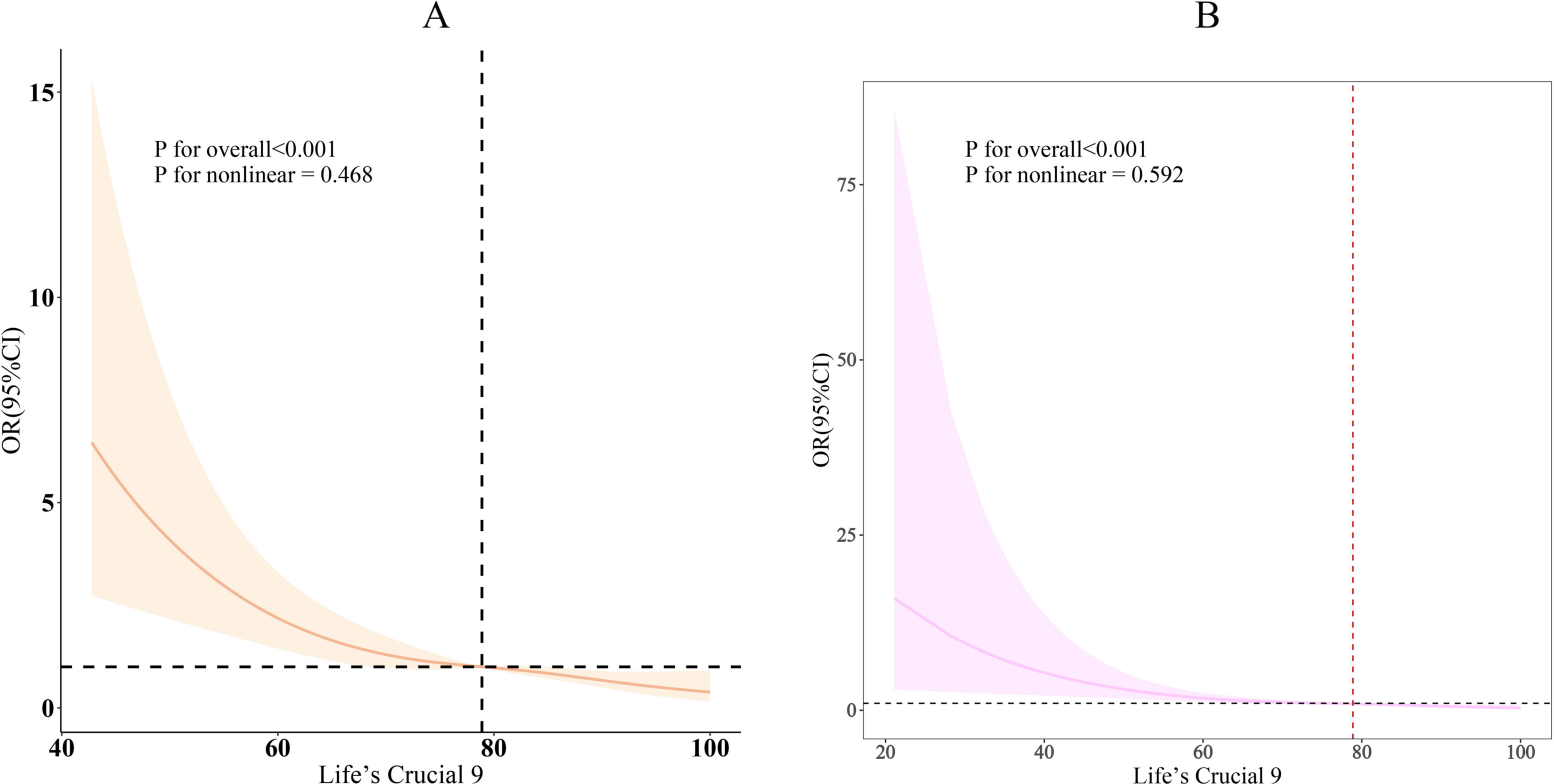
Dose-response relationships between LC9 and infertility. **(A)** weighted; **(B)** unweighted. OR (solid lines) and 95% confidence levels (shaded areas) were adjusted for age, education level, marital, PIR, race, smoking, drinking, Physical activity, hypertension, diabetes, and high cholesterol.

As shown in **Table 2**, three different models were used to assess the association between LC9 and its nine sub-scores with infertility. In Model 3, after full adjustment for covariates, each 10-point increase in LC9 was associated with a 35% reduction in the odds of infertility [odds ratio: 0.65 (95% confidence interval: 0.54, 0.80)]. As the LC9 quartiles increased (with Q1 as the reference), the incidence of infertility progressively decreased, with the corresponding results being: Q2: [odds ratio: 0.53 (95% confidence interval: 0.32, 0.87)], Q3: [odds ratio: 0.33 (95% confidence interval: 0.17, 0.62)], and Q4: [odds ratio: 0.27 (95% confidence interval: 0.15, 0.50)]. Additionally, a statistically significant trend was observed with lower infertility incidence as LC9 increased (P for trend < 0.001). In all models, except for the physical activity score, other LC9 sub-scores maintained a significant negative association with infertility. Furthermore, consistent results were obtained in the unweighted analysis (**Supplementary Table S5**). **Figure 2** further illustrates the significant negative correlation between LC9 and the prevalence of infertility (P for overall < 0.001; P for non-linear = 0.468).

**Table 2:** Adjusted odds ratios (ORs) of LC9 and infertility, weighted.

LC9 components (per 10 scores)	Model 1 [OR (95% CI)]	*P*	Model 2 [OR (95% CI)]	*P*	Model 3 [OR (95% CI)]	*P*
**Total LC9 score**	0.74 (0.65, 0.83)	**<0.001**	0.72 (0.64, 0.82)	**<0.001**	0.65 (0.54, 0.80)	**<0.001**
Quartile						
Q1	1 (ref.)		1 (ref.)		1 (ref.)	
Q2	0.62 (0.43, 0.89)	**0.010**	0.58 (0.39, 0.87)	**0.009**	0.53 (0.32, 0.87)	**0.014**
Q3	0.46 (0.30, 0.72)	**<0.001**	0.41 (0.26, 0.65)	**<0.001**	0.33 (0.17, 0.62)	**0.001**
Q4	0.33 (0.20, 0.55)	**<0.001**	0.31 (0.19, 0.53)	**<0.001**	0.27 (0.15, 0.50)	**<0.001**
***P for trend***	<0.001		<0.001		<0.001	
Subgroup LC9 scores						
HEI diet score	0.95 (0.90, 1.00)	**0.046**	0.94 (0.89, 0.99)	**0.017**	0.95 (0.90, 1.01)	0.100
Physical activity score	0.97 (0.93, 1.01)	0.140	0.98 (0.93, 1.02)	0.130	1.11 (0.94, 1.32)	0.200
Tobacco exposure score	0.96 (0.93, 0.99)	**0.028**	0.96 (0.92, 0.99)	**0.024**	0.98 (0.78, 1.32)	>0.9
Sleep health score	0.95 (0.90, 0.99)	**0.041**	0.92 (0.88, 0.98)	**0.005**	0.92 (0.85, 0.99)	**0.026**
Psychological health	0.93 (0.89, 0.98)	**0.009**	0.91 (0.86, 0.95)	**<0.001**	0.92 (0.87, 0.97)	**0.006**
Body mass index score	0.92 (0.87, 0.96)	**<0.001**	0.92 (0.87, 0.97)	**0.003**	0.92 (0.86, 0.98)	**0.010**
Blood lipid score	0.93 (0.88, 0.99)	**0.014**	0.96 (0.90, 1.02)	0.200	0.98 (0.90, 1.05)	0.500
Blood glucose score	0.86 (0.81, 0.92)	**<0.001**	0.89 (0.83, 0.95)	**0.001**	0.85 (0.74, 0.98)	**0.029**
Blood pressure score	0.90 (0.85, 0.96)	**0.001**	0.92 (0.86, 0.98)	**0.015**	0.92 (0.82, 1.02)	0.100

Model 1: no covariates were adjusted.

Model 2: age, education level, marital, PIR, and race were adjusted.

Model 3: age, education level, marital, PIR, race, smoking, drinking, Physical activity, hypertension, diabetes, and high cholesterol were adjusted.

LC9, Life’s Crucial 9; PIR, Ratio of family income to poverty; ORs, odds ratios; CI, confidence interval.

The bold values are less than 0.05.

### 3.3 Subgroup analysis

**Figure 3:**
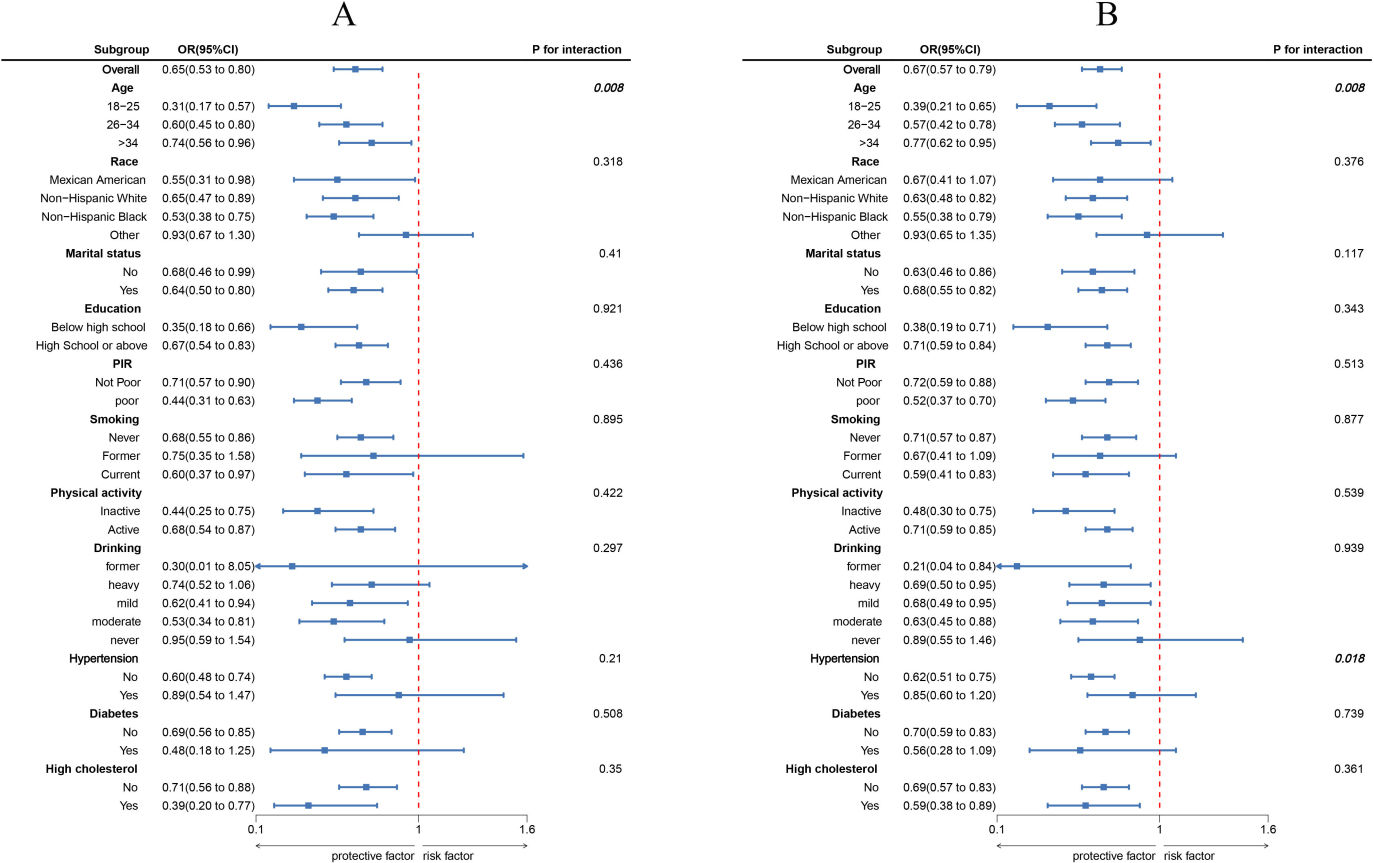
Subgroup analysis between LC9 and infertility. **(A)** weighted; **(B)** unweighted. ORs were calculated as per 10-unit increase in LC9. Analyses were adjusted for age, education level, marital, PIR, race, smoking, drinking, Physical activity, hypertension, diabetes, and high cholesterol.

The results of the subgroup analysis are shown in **Figure 3**. In most subgroups, LC9 scores were negatively correlated with infertility (P < 0.05). There was a significant interaction between LC9 scores and age (P < 0.05). For every 10-point increase in LC9, the odds of infertility decreased by 69% [0.31 (0.17, 0.57)] in women aged 18-25, which was significantly higher than the reduction observed in women aged 26-34 [0.60 (0.45, 0.80)] and those aged 35-45 [0.74 (0.56, 0.96)]. Similarly, in the unweighted subgroup analysis shown in **Figure 3B**, there were significant interactions between LC9 scores and both age and blood pressure (P < 0.05).

### 3.4 WQS analyses

**Figure 4:**
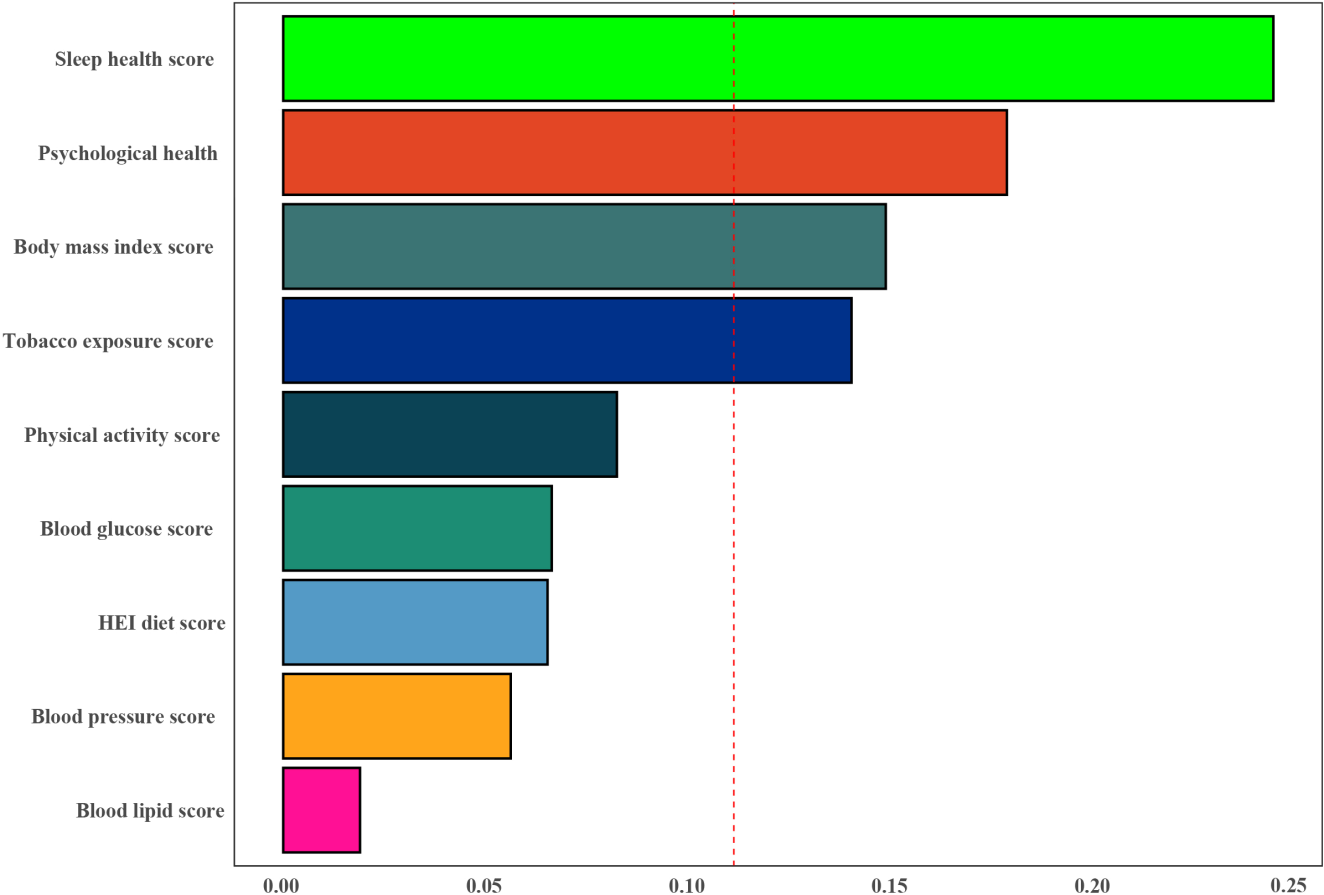
Weights represent the proportion of partial effect for each LC9 metric in the WQS regression. The model adjusted for age, education level, marital status, PIR, and race.

The WQS index from the WQS regression was negatively associated with the risk of infertility (OR 0.27,95% CI 0.14 to 0.53) (**Supplementary Table S6**). **Figure 4** showed that all sub-scores were negatively associated with infertility, with sleep health (weight = 0.244) identified as the most important factor influencing the presence of infertility, followed by psychological health and Body mass index score (weights = 0.178 and 0.148).

## 4 Discussion

In this study, we found a significant negative correlation between CVH (quantified by LC9 scores) and the risk of infertility. Subgroup analysis further revealed a more pronounced interaction between LC9 scores and age in women younger than 35 years. This underscores the potential impact of cardiovascular health on the occurrence of infertility and highlights the importance of early monitoring and control of CVH, as quantified by LC9 scores, to reduce the incidence of infertility. In addition, the WQS regression results showed that sleep health (weight = 0.244) was considered the most important factor influencing the occurrence of infertility, followed by mental health and body mass index score (weights = 0.178 and 0.148).

To our knowledge, this is the first study to investigate the association between the new CVH metric (LC9) and the prevalence of infertility. Previous research has indicated that women with infertility have an increased risk of poorer CVH in middle age (quantified by LE8 scores) compared to women without infertility (13). This suggests a link between infertility and cardiovascular health, providing valuable insights for exploring CVH metrics and their relationship with infertility.

However, merely indicating the association between LE8 and infertility is insufficient. Infertility is a multifactorial condition closely related to various cardiovascular risk factors. For instance, a cross-sectional study involving 1,820 participants highlighted a strong association between sleep disorders and female infertility, potentially linked to the activation of the HPA axis (hypothalamic-pituitary-adrenal axis), which reduces endometrial receptivity (27). Additionally, a prospective study involving 385,292 participants demonstrated that healthy sleep patterns are associated with a reduced risk of cardiovascular diseases, coronary heart disease, and stroke (28). Another case-control study of 180 infertile women and 540 fertile women indicated that the psychological health of infertile women might be adversely affected (29). These shared risk factors may underlie both infertility and cardiovascular disease, with women experiencing infertility having a higher prevalence of cardiovascular risk factors even before conception (30). These pieces of evidence suggest that the CVH score assessed by LC9 could serve as an indicator of both cardiovascular health status and infertility incidence. Our study results demonstrate a negative linear relationship between CVH and infertility from various perspectives. For example, WQS results indicate that sleep health, mental health, BMI, and tobacco exposure are more closely associated with infertility after adjusting for other confounding factors.

The mechanisms underlying the relationship between cardiovascular health and infertility are multifaceted and complex. Possible mechanisms include:(1) Insulin Resistance (IR): Insulin resistance is a pathogenic mechanism in various diseases. Research suggests that IR may affect oocyte quality by reducing mitochondrial function and exacerbating oxidative stress in follicles, thereby further impairing mitochondrial function and inducing the release of inflammatory factors (31). (2) Lipid Metabolism Disorders: Dyslipidemia, a major contributor to cardiovascular disease, is typically characterized by elevated circulating levels of low-density lipoprotein cholesterol (LDL-C) and/or triglycerides (TG), along with reduced levels of high-density lipoprotein cholesterol (HDL-C). Women with lipid metabolism disorders tend to exhibit deteriorated cumulus-oocyte complex (COC) morphology and a decreased number of cleavage-stage embryos. Elevated TC concentrations may adversely affect endometrial receptivity, leading to infertility (8). (3) Endothelial Dysfunction: It is well-known that the incidence of cardiovascular diseases is significantly lower in premenopausal women compared to men, largely due to the protective role of estrogen on cardiovascular health. Estrogen regulates lipoproteins and possesses anti-apoptotic properties by increasing the activation of the protective PI3K/Akt pathway and reducing inflammation and cytokine production. Endothelial cell damage is closely related to cardiovascular diseases, and estrogen also plays a role in vascular function regulation. Studies suggest that the decline in regulatory function is due to estrogen loss rather than aging (32). Endocrine abnormalities causing female infertility, such as polycystic ovary syndrome (PCOS), are associated with endocrine disorders like hyperandrogenism.

Studies have shown that sleep disorders, including disturbances in sleep duration and continuity, can interfere with reproductive capabilities or lead to the activation of the hypothalamic-pituitary-adrenal (HPA) axis, further disrupting reproductive functions. Sleep disorders may also affect reproductive hormones such as thyroid-stimulating hormone (TSH), follicle-stimulating hormone (FSH), and prolactin (PRL) (3). Age is negatively correlated with oocyte quality, potentially due to the accumulation of DNA damage in oocytes from environmental stress factors over time or the first-in-first-out principle, where higher-quality oocytes are ovulated earlier in life. Additionally, the shortening of oocyte telomeres with age contributes to the decline in oocyte and embryo viability (33). A meta-analysis indicated that psychological interventions could improve pregnancy rates (20). For example, a study by Rong Zhou and colleagues (34) demonstrated that cognitive behavioral therapy (CBT) and the integrative body–mind–spirit (BMS) approach play important roles in improving pregnancy rates, with BMS being associated with reduced anxiety. Additionally, a prospective, controlled, single-blind, randomized study by Alice D. Domar et al. (35), which included 184 women who had been attempting to conceive for 1 to 2 years, demonstrated that group psychological interventions appeared to increase pregnancy rates in women with infertility. Moreover, factors such as obesity, diet, smoking (33), and lipid abnormalities—components of LC9—affect these pathophysiological processes. Therefore, these studies collectively emphasize the importance of LC9 in infertility and provide further theoretical support for this research.

The results of this study provide important guidance for the management and prevention of infertility. This is the first time LC9 has been used to predict the risk of infertility, demonstrating its potential clinical value. By adjusting for other confounding factors, the results are more reliable, and the representative sample obtained through national sampling allows for extrapolation to a larger population. However, there are limitations: (1) This study is cross-sectional, which prevents establishing a causal relationship between LC9 and infertility (36). Large-scale cohort studies are needed to further validate the current findings. (2) Data collection primarily relied on questionnaires, which may introduce measurement errors. (3) Despite adjusting for many confounding factors, the limitations of the NHANES database prevented us from including certain potential confounders of infertility, such as male-only causes, uterine structural abnormalities, and other anatomical factors, which may have a weaker association with CVH. (4) This study did not take into account participants’ job status or their exposure to other chemical and physical pollutants. For example, global warming and working in hot and humid environments may impact reproductive health in both men and women, potentially leading to infertility, which could affect the study’s findings. Therefore, caution should be exercised when interpreting the results due to this limitation. In future studies, we plan to include these factors as confounding variables in the model to improve the accuracy of infertility research.

## 5 Conclusion

In conclusion, the findings of this study reveal a significant protective association between Life’s Crucial 9 (LC9) and the incidence of infertility. This discovery not only provides scientific support for the application of LC9 in infertility risk assessment but also underscores its potential clinical significance in promoting reproductive health. By integrating LC9 into routine health management, healthcare professionals can more effectively identify and manage high-risk populations for infertility, thereby enhancing overall reproductive health. Furthermore, this study lays the groundwork for future research to validate the efficacy and mechanisms of LC9 in larger populations, advancing the development of infertility prevention strategies.

## Data Availability

The datasets presented in this study can be found in online repositories. The names of the repository/repositories and accession number(s) can be found in the article/**Supplementary Material**.
